# Supplementary material for: The interactome of intact mitochondria by cross-linking mass spectrometry provides evidence for coexisting respiratory supercomplexes
Source: Mol Cell Proteomics. 2017 Dec 8;17(2):216–32. doi: 10.1074/mcp.RA117.000470 (PMC5795388; doi:10.1074/mcp.RA117.000470)
Supplement: Supplemental Data [file supp_RA117.000470_133922_0_supp_23978_fzffwf.zip › spectra_annotation/mito_spectra_annotation/312-1-3-1-17-1.pdf]

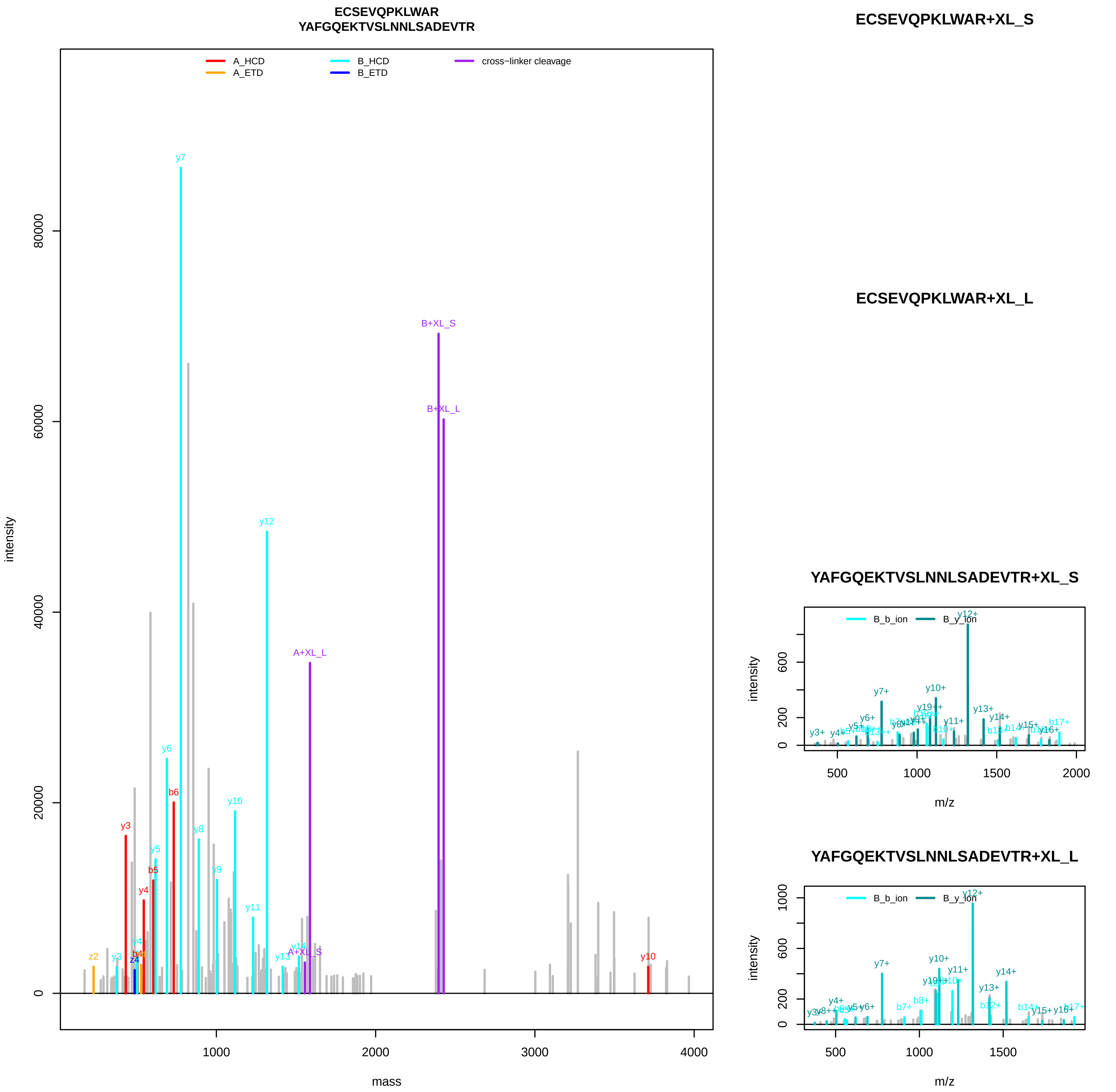

ECSEVQPKLWAR
YAFGQEKTVSLNNLSADEVTR
A_HCD
A_ETD
B_HCD
B_ETD
cross−linker cleavage
intensity
mass
0
20000
40000
60000
80000
1000
2000
3000
4000
y7
B+XL_S
B+XL_L
y12
A+XL_L
A+XL_S
y6
b6
y3
y4
b5
y5
y8
y9
y10
y11
y13
y14
z2
z4
y10
ECSEVQPKLWAR+XL_S
ECSEVQPKLWAR+XL_L
YAFGQEKTVSLNNLSADEVTR+XL_S
YAFGQEKTVSLNNLSADEVTR+XL_L
B_b_ion
B_y_ion
m/z
500
1500
0
200
600
1000
y12+
y10+
y7+
y13+
y14+
y11+
y15+
y16+
b17+
